# Changes in food choices and dietary patterns during the lifestyle intervention and their association with type 2 diabetes risk in participants with high or low genetic risk for type 2 diabetes

**DOI:** 10.1007/s00394-025-03791-x

**Published:** 2025-09-11

**Authors:** Ulla Tolonen, Maria Lankinen, Markku Laakso, Ursula Schwab

**Affiliations:** 1 Institute of Public Health and Clinical Nutrition, University of Eastern Finland, PO Box 1627, 70211 Kuopio, Finland; 2 Institute of Clinical Medicine, Internal Medicine, University of Eastern Finland, Kuopio, Finland; 3 Department of Medicine, Kuopio University Hospital, Kuopio, Finland; 4 Department of Medicine, Endocrinology and Clinical Nutrition, Kuopio University Hospital, Kuopio, Finland

**Keywords:** Diet, Human, Diabetes, Clinical trial, Lifestyle, Gene

## Abstract

**Purpose:**

To investigate how a group-based lifestyle intervention affects food choices and if the dietary patterns at the end of the intervention are associated with incidence type 2 diabetes (T2D). We also investigated if the possible associations between diet and T2D risk were modified by the genetic risk for T2D.

**Methods:**

Participants in the T2D-GENE study were men with prediabetes aged 50–75 years, body mass index  ≥ 25 kg/m^2^, belonging in either low or high genetic risk score (GRS) tertile for T2D. They participated in a 3 year, group-based T2D-GENE lifestyle study (either an intervention or a control arm). Food consumption was measured with a food frequency questionnaire (FFQ) at baseline and at year 3. We included in our study all the T2D-GENE participants who had FFQ available at year 3 (*n* = 883). To diagnose T2D we used the following criteria, fasting plasma glucose  ≥ 7.0 mmol/l, 2 h plasma glucose  ≥ 11.1 mmol/l, or HbA1C  ≥ 48 mmol/mol ( ≥ 6.5%). The GRS was based on 76 genetic variants associated with T2D.

**Results:**

There were statistically significant changes towards more recommended food consumption (higher frequency of whole-grain products, vegetables, and non-tropical vegetable oils) in the participants receiving lifestyle counselling as compared to their baseline and to the population controls. The intervention group reported increased consumption of healthy dietary pattern (high in e.g. vegetables, whole-grain products, and fish) and decreased consumption of unhealthy (high in e.g. meat, sausages and low-fibre products) at year three as compared to baseline. End-of-intervention healthy dietary pattern was associated with a decrease in the risk of T2D (OR 0.67, 95% CI 0.46; 0.97 in multivariable model) and end-of-intervention unhealthy pattern with increased risk (OR 1.82, 95% CI 1.26; 2.62 in multivariable model). When stratified by the GRS, the associations remained significant for the high genetic risk group.

**Conclusion:**

A group-based lifestyle intervention improved diet quality. Healthy dietary pattern associated with lower risk for T2D whereas unhealthy pattern associated with higher risk. After stratification by the GRS, associations were evident in participants with a high genetic risk for T2D.

**Supplementary Information:**

The online version contains supplementary material available at 10.1007/s00394-025-03791-x.

## Introduction

Type 2 diabetes (T2D) is a globally increasing health problem [1]. T2D is caused by both genetic factors and lifestyle, and it can be effectively prevented or delayed by lifestyle choices, such as healthy diet [2–5].

Many lifestyle intervention studies on the prevention of T2D to date have focused on individual dietary counselling with [3, 6, 7] or without group-based approaches [2, 8–10]. These prevention studies have been carried out in individuals having impaired glucose tolerance (IGT) [2, 3, 6–8, 10]. Only some have included patients with isolated impaired fasting glucose (IFG) [9, 11–14] or focused on group-based approaches [11–13, 15]. Individual dietary counselling is resource-intensive and, therefore, modern approaches demanding less health care resources are needed.

Also, the evidence on the interaction between genes and diet in the prevention of T2D is still limited and contradictory and mostly based on epidemiological studies. Some previous studies suggest that there is no interaction between the diet and genetic T2D burden and that everyone, regardless of their genetic risk for T2D, benefits from healthy lifestyle equally [16–19]. However, there are also previous studies reporting an interaction between the diet and genetic risk of T2D [20–24].

We have previously studied the impact of dietary pattern and genetic interactions in a cross-sectional setting showing that there was no interaction between the diet and genetic risk of T2D. Healthy dietary pattern was associated with a low risk for hyperglycemia both in the participants with a low and high genetic risk for T2D [25]. Our 3 year T2D-GENE intervention study [26] showed that a group-based lifestyle intervention was successful in preventing T2D and improving dietary quality (the consumption of vegetables, fruits and berries, amount of dietary fibre, and quality of fat) among those in the intervention group receiving lifestyle counselling, also in participants with a high genetic risk for T2D. The dietary comparisons between the intervention and control groups, or within the control group have not yet, however, been performed.

In the current study, we investigated how a 3 year group-based lifestyle intervention in the T2D-GENE study, also utilising a web portal, affects food choices and whether there are differences between the intervention and control groups at the end of the intervention. We also evaluated the association of healthy and unhealthy dietary patterns with the risk of T2D and if the genetic risk for T2D modifies the risk. This is the first study to investigate the changes in food choices in the T2D-GENE study as compared to the control group, and the first analysis from the T2D-GENE study investigating how healthy and unhealthy dietary patterns and genetic risk score interact in the development of T2D.

## Methods

### Study participants

The intervention and control group of the T2D-GENE study were recruited from the Metabolic Syndrome in Men (METSIM) cohort [27]. Inclusion criteria in T2D-GENE study were age 50–75 years, IFG with or without IGT (fasting plasma glucose (FPG) 5.6–6.9 mmol/l, 2 h plasma glucose (PG) < 11.0 mmol/l, haemoglobin A1c (HbA1c) < 48 mmol/mol (< 6.5%)), body mass index (BMI)  ≥ 25.0 kg/m^2^, and genetic risk score (GRS) in either the lowest or the highest tertile. People with the medium GRS tertile were excluded. A total of 628 subjects met the inclusion criteria and were willing to participate based on invitations from the METSIM cohort. They were allocated to the intervention group. A total of 14 participants dropped out after the first group session and are thus excluded from the number of participants who received the allocated intervention. The control group was composed in a non-randomised manner from a matching population continuing with the METSIM study protocol including an extra laboratory visit with a 2 h oral glucose tolerance test (OGTT) at year 3. The control group was matched with the intervention group by their GRS for T2D, age, BMI and FPG. A total of 345 controls were willing to have a laboratory visit in year 3. The participant recruitment and the study arms of the T2D-GENE study have been described in more detail earlier [26, 28].

**Fig. 1:**
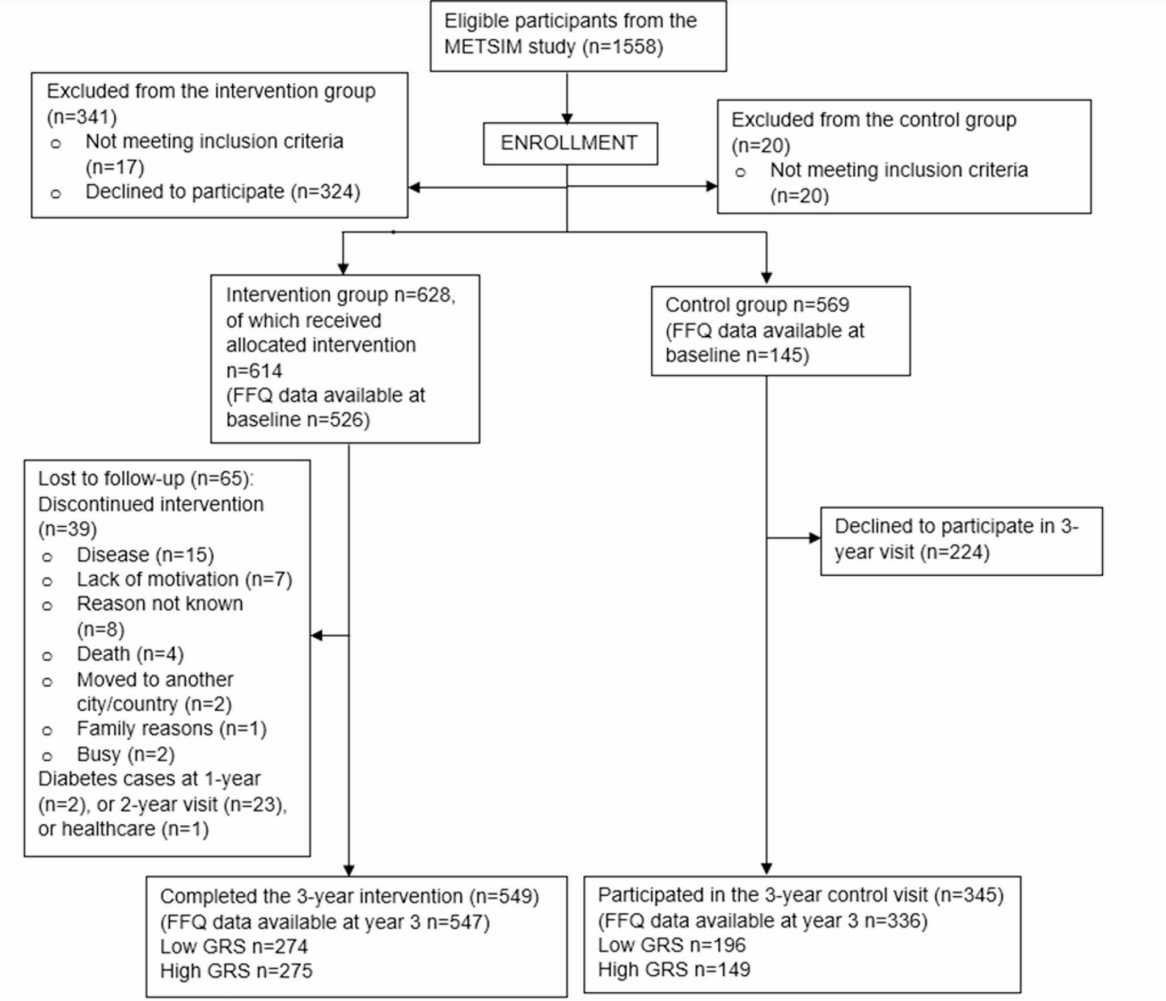
Participant flow chart of the T2D-GENE study, modified from Lankinen et al. [25]. FFQ = Food frequency questionnaire, GRS = Genetic risk score

In the current study we have included all the participants of the T2D-GENE study having food frequency questionnaire (FFQ) data available. Due to some missing data from different timepoints, the study population sizes are as follows, *n* = 605 in paired analyses comparing baseline and year 3 (*n* = 465 and *n* = 140 for the intervention and the control groups, respectively) and *n* = 883 for year 3 data only (*n* = 547 and *n* = 336 for intervention and control groups, respectively) (Fig. 1).

A total of 84.5% of the study participants had isolated IFG and 15.5% combined IFG and IGT. The participants in the intervention group were significantly older after false discovery rate (FDR) adjustment than those in the control group (Table 1). The food consumption frequencies differed at baseline for low-fibre porridges, fresh and frozen berries, and for the overall healthy dietary pattern, the intervention group consuming more frequently berries and overall healthy diet, and the control group consuming more low-fibre porridges. Otherwise, the groups had a similar diet at baseline.

**Table 1:** Baseline characteristics of the study participants

	Intervention group (*n* = 547)	Control group (*n* = 336)	*p*-value
Age, years	65.1 (5.9)	63.8 (5.5)	0.002^a†^
Height, cm	176.6 (6.1)	176.6 (6.7)	0.991^a^
Weight, kg	89.1 (11.1)	88.7 (11.2)	0.576^a^
Body mass index, kg/m^2^	28.6 (3.0)	28.4 (3.0)	0.517^a^
Waist-hip-ratio	1.0 (0.1)	1.0 (0.1)	0.671^a^
Total cholesterol, mmol/l	5.0 (1.0)	5.0 (1.1)	0.991^a^
LDL cholesterol, mmol/l	3.1 (0.9)	3.1 (0.9)	0.971^a^
HDL cholesterol, mmol/l	1.4 (0.4)	1.4 (0.4)	0.371^a^
Triglycerides, mmol/l	1.3 (0.6)	1.3 (0.6)	0.599^a^
Systolic blood pressure, mmHg	133 (15)	135 (16)	0.434^a^
Diastolic blood pressure, mmHg	84 (9)	84 (10)	0.654^a^
Total alcohol consumption, g/week^1^	78.8 (113.9)	77.7 (94.3)	0.912^a^
Smoking current, smokers, %	7.9	11.6	0.031^b^
Leisure time exercise level, physically active, %	72.9	69.6	0.325^b^

Values are mean values with standard deviations unless stated otherwise. ^1^Intervention *n* = 339, ^1^control *n* = 176. ^a^Independent samples t-test, ^b^Chi-square test for independence. ^†^Statistically significant after false discovery rate adjustment

**Table 2:** Scores (1–8) for food consumption frequency of the intervention and the control groups at baseline and the end of intervention (year 3)

	Intervention group (*n* = 465)			Control group (*n* = 140)			
Food	Baseline, mean (SD)	Year 3, mean (SD)	*p*-value (change over time)^2^	Baseline, mean (SD)	Year 3, mean (SD)	*p*-value (change over time)^2^	*p*-value (difference between groups at year 3)^3^
*Cereals*							
Buns, bun-based pies	3.77 (1.61)	3.49 (1.46)	< 0.001^†^	3.74 (1.67)	3.69 (1.49)	0.651	< 0.001^†^
Sweet cookies, biscuits	3.01 (1.43)	2.68 (1.40)	< 0.001^†^	3.06 (1.43)	3.06 (1.44)	1.000	< 0.001^†^
Other sweet pastries (e.g. cakes, Danish pastry)	2.09 (0.92)	1.85 (0.85)	< 0.001^†^	2.16 (1.02)	2.06 (0.82)	0.285	0.001^†^
Savoury pies and pastries (e.g. Carelian pie)	3.06 (1.22)	2.73 (1.14)	< 0.001^†^	3.21 (1.09)	2.95 (1.06)	0.010	< 0.001^†^
Pizza	1.57 (0.63)	1.49 (0.60)	0.006^†^	1.60 (0.62)	1.59 (0.61)	0.900	0.049
Hamburgers	1.27 (0.51)	1.20 (0.45)	0.006^†^	1.30 (0.51)	1.28 (0.55)	0.656	0.093
Refined pasta or rice	2.37 (1.00)	2.09 (0.95)	< 0.001^†^	2.34 (0.90)	2.33 (0.84)	0.847	< 0.001^†^
Whole grain pasta or rice	1.96 (0.92)	2.15 (0.94)	< 0.001^†^	1.91 (0.88)	1.91 (0.86)	1.000	0.005^†^
Low-fibre porridges (e.g. rice and semolina porridges)*	1.71 (0.90)	1.61 (0.82)	0.023^†^	2.13 (1.30)	1.83 (1.90)	0.018	< 0.001^†^
Whole grain porridges (e.g. from oat, rye, or from mixture of oat, rye, barley and wheat)	3.71 (1.76)	4.12 (1.73)	< 0.001^†^	3.29 (1.72)	3.59 (1.80)	0.047	< 0.001^†^
Breakfast cereals and muesli	1.97 (1.55)	2.17 (1.66)	0.009^†^	1.79 (1.40)	1.79 (1.33)	1.000	0.143
Rye or crisp bread	5.76 (1.52)	5.73 (1.42)	0.663	5.78 (1.50)	5.51 (1.56)	0.038	0.052
Yeast bread, graham and whole grain breads including buns and toasts	3.92 (1.85)	3.58 (1.76)	< 0.001^†^	3.86 (1.72)	3.76 (1.72)	0.506	0.032^†^
French roll, baguette, or other white bread	1.80 (1.15)	1.59 (0.98)	< 0.001^†^	2.04 (1.31)	1.89 (1.21)	0.244	< 0.001^†^
*Dairy products*							
Unsweetened or artificially sweetened yoghurt^1^, quark, or Nordic sour milk (> 1% fat)	2.17 (1.56)	1.96 (1.44)	0.023^†^	2.36 (1.63)	2.20 (1.57)	0.297	0.013^†^
Unsweetened or artificially sweetened yoghurt^1^, quark, Nordic sour milk, or skyr (≤ 1% fat)	2.25 (1.62)	2.27 (1.61)	0.814	2.19 (1.50)	2.04 (1.33)	0.265	0.094
Sweetened yoghurt^1^, quark, or Nordic sour milk (> 1% fat)	2.25 (1.56)	1.85 (1.33)	< 0.001^†^	2.31 (1.60)	2.29 (1.47)	0.923	< 0.001^†^
Sweetened yoghurt^1^, quark, Nordic sour milk, or skyr (≤ 1% fat)	1.84 (1.27)	1.85 (1.33)	0.976	1.83 (1.22)	1.69 (1.06)	0.246	0.616
Low-fat cheeses (fat ≤ 17%, e.g. Edam 17, Oltermanni 17, Polar 10)	4.31 (1.93)	4.51 (1.78)	0.027^†^	4.36 (1.87)	3.78 (1.95)	< 0.001^†^	< 0.001^†^
Cheeses with > 17% fat (e.g. Edam, Emmental, Aura, Brie)	2.98 (1.85)	2.03 (1.31)	< 0.001^†^	2.94 (1.67)	2.71 (1.81)	0.112	< 0.001^†^
Ice cream or puddings	2.13 (1.05)	2.01 (1.01)	0.008^†^	2.14 (1.07)	2.06 (0.88)	0.355	0.018^†^
*Potato, Vegetables*							
Boiled or mashed potatoes	4.10 (1.10)	4.11 (1.02)	0.927	3.88 (1.01)	3.98 (0.90)	0.245	0.070
Fried potatoes or French fries	1.77 (0.84)	1.70 (0.84)	0.045	1.79 (0.78)	1.71 (0.79)	0.245	0.422
Vegetable dishes (e.g. soups, casseroles, stews)	3.29 (1.02)	3.22 (1.03)	0.156	3.14 (1.13)	3.01 (1.11)	0.245	0.056
Boiled side vegetables	3.32 (1.30)	3.30 (1.25)	0.730	3.20 (1.16)	3.14 (1.23)	0.581	0.019^†^
Fresh salad, fresh vegetables	5.17 (1.31)	5.17 (1.09)	0.971	4.82 (1.37)	4.74 (1.31)	0.430	< 0.001^†^
*Salad dressings*							
Oil-based salad dressing or oil with vegetables	3.35 (1.78)	3.47 (1.80)	0.151	3.27 (1.77)	3.13 (1.69)	0.301	0.001^†^
Sour cream-based salad dressing	1.79 (1.05)	1.55 (0.96)	< 0.001^†^	1.87 (1.11)	1.59 (0.88)	0.002^†^	0.017^†^
Non-fatty salad dressing, e.g. fruit juice	1.47 (0.94)	1.39 (0.87)	0.134	1.73 (1.16)	1.53 (0.96)	0.061	0.170
*Fruits, Berries*							
Fruits	5.14 (1.25)	5.24 (1.13)	0.083	4.82 (1.36)	4.67 (1.33)	0.159	< 0.001^†^
Fresh or frozen berries*	4.19 (1.62)	4.35 (1.47)	0.012^†^	3.76 (1.42)	3.65 (1.41)	0.315	< 0.001^†^
Fruit or berry juices (no added sugar)	2.98 (1.83)	2.59 (1.71)	< 0.001^†^	3.29 (1.73)	2.80 (1.64)	0.002^†^	0.190
*Fish*							
Fish and fish dishes in total	3.40 (0.95)	3.44 (0.79)	0.378	3.18 (0.84)	3.13 (0.87)	0.427	< 0.001^†^
Rainbow trout, salmon, arctic char, mackerel (e.g. fried, in a soup)	2.72 (0.90)	2.88 (0.86)	< 0.001^†^	2.67 (0.92)	2.62 (0.85)	0.543	< 0.001^†^
Baltic herring (e.g. patties, smoked)	1.33 (0.55)	1.25 (0.52)	0.005^†^	1.26 (0.49)	1.24 (0.52)	0.624	0.904
Vendace, bream, whitefish	1.86 (0.84)	1.75 (0.76)	0.005^†^	1.72 (0.81)	1.64 (0.72)	0.164	0.016^†^
Other fish (e.g. pike, perch, pike perch, saithe, cod)	2.23 (1.02)	2.14 (1.00)	0.032^†^	2.03 (0.92)	2.09 (0.94)	0.358	0.514
*Meat, Sausage, Egg*							
Meat dishes (e.g. roasts, minced meat sauce, steaks)	3.36 (1.04)	3.01 (1.00)	< 0.001^†^	3.32 (1.05)	3.19 (1.03)	0.253	0.002^†^
Chicken, turkey, and chicken dishes	3.29 (0.97)	3.40 (0.92)	0.007^†^	3.21 (1.01)	3.16 (0.99)	0.609	0.003^†^
Sausage dishes, sausages	2.57 (1.00)	2.27 (0.91)	< 0.001^†^	2.68 (1.23)	2.55 (1.02)	0.129	< 0.001^†^
Sausage cutleries (e.g. mettwurst, bologna sausage)	2.89 (1.80)	1.87 (1.34)	< 0.001^†^	3.04 (1.79)	2.79 (1.77)	0.082	< 0.001^†^
Whole meat cuts (e.g. ham, turkey)	4.52 (1.74)	4.52 (1.65)	0.978	4.51 (1.45)	4.24 (1.60)	0.064	0.086
Eggs (boiled, fried, omelets)	3.23 (1.16)	3.27 (1.04)	0.379	3.19 (1.12)	3.16 (1.11)	0.756	0.391
*Other*							
Chocolate	2.38 (1.18)	2.21 (1.15)	< 0.001^†^	2.34 (1.10)	2.43 (1.10)	0.241	0.108
Other candy	2.37 (1.22)	2.05 (1.17)	< 0.001^†^	2.14 (1.03)	2.21 (1.20)	0.413	0.006^†^
Savory snacks (e.g. chips, popcorn)	1.39 (0.70)	1.25 (0.55)	< 0.001^†^	1.49 (0.81)	1.38 (0.75)	0.161	< 0.001^†^
Ready-meals	1.99 (1.04)	1.98 (1.04)	0.806	2.05 (1.24)	1.96 (1.05)	0.307	0.229

^1^including dairy-, oat-, soy- and rice-based products, ^2^paired samples *t*-test, ^3^independent samples *t*-test (intervention group *n* = 547, control group *n* = 336), *statistically significant difference between intervention and control group at baseline (with false discovery rate adjustment), ^†^statistically significant after false discovery rate adjustment

### Intervention

The intervention group in the T2D-GENE lifestyle intervention participated in 5–7 group sessions (the additional two sessions focusing on body weight management for those having baseline BMI > 28 kg/m^2^). The sessions were organised throughout a 3-year period: sessions 1–3, or 1–5, within the first months and following sessions at year 1 and 2. Participation rate for the sessions targeted to all intervention group participants was 90.7–99.5%, and 53.8% and 55.6% for sessions 4 and 5 aimed at participants with BMI > 28 kg/m^2^, respectively [29]. An additional web portal was created for delivering online material and serving as a discussion platform for the intervention group. The intervention group had laboratory visits at year 0, 1, 2 and 3. Online materials were delivered monthly during the 3-year period. The control group only had laboratory visits at baseline and year 3. They had no group sessions. The control group was only given general lifestyle counselling by study nurses at baseline laboratory visit, as a part of the METSIM cohort protocol.

Dietary targets for the intervention group were based on the Finnish and the Nordic nutrition recommendations with special focus on improving the quality of dietary fat and carbohydrates, and increasing consumption of vegetables, fruits and berries. The lifestyle intervention T2D-GENE study has been described in more detail earlier [26, 28, 29]. The T2D-GENE study was registered at ClinicalTrials.gov (ID: NCT02709057).

### Dietary assessment

We assessed diet using a 47-point self-administered qualitative FFQ at baseline and at the end of the 3-year intervention (Online Resource 1, Supplementary Information 1). The FFQ used was a slightly modified and updated version from the FFQ used in the basic questionnaire of the Finrisk 2007 National Health Survey [30] and with a few additional questions as compared to our previous study [25]. Based on the questionnaire, each participant was given a score of 1 to 8 based on the consumption frequency for each food surveyed (“1” for “consumption less than once per month or never” up to “8” for “over 4 times per day”, or “1” for “less than 1 slice per day or none” up to “8” for “6 or more slices per day” for breads). We also collected data on typical spread for bread and fat used for cooking using multiple choice questions. Registered nurses or clinical nutritionists were available for instructing and assisting with the filling of the FFQ during a study visit.

### Dietary patterns

In addition to changes in the consumption of individual foods, we analyzed the changes for two dietary patterns, healthy and unhealthy. These were created by principal component analysis in our previous cross-sectional study; the pattern creation process has been described earlier [25]. The healthy dietary pattern consists of fresh salad, fresh vegetables; fresh or frozen berries; boiled side vegetables; fruits; oil-based salad dressing or oil with vegetables; fish and fish dishes; chicken, turkey and chicken dishes; unsweetened or artificially sweetened yoghurt (including dairy-, oat-, soy- and rice-based products), quark, Nordic sour milk, or skyr (≤ 1% fat); vegetable dishes; whole grain porridges; whole grain pasta or rice; low-fat cheeses (fat ≤ 17%); boiled or mashed potatoes. The unhealthy pattern consists of fried potatoes or French fries; sausage dishes, sausages; hamburgers; pizza; refined pasta or rice; other sweet pastries; sausage cutleries; other candy; savory pies and pastries; savory snacks; ice cream or puddings; French roll, baguette, or other white bread; sweet cookies, biscuits; meat dishes; ready-meals; cheeses with > 17% fat; sweetened yoghurt (including dairy-, oat-, soy- and rice-based products), quark, or Nordic sour milk (> 1% fat); sour cream-based salad dressing. We calculated a point sum for the foods included in each pattern at baseline and at year three for each participant. For T2D risk assessment, we further created tertiles of year three consumption of both dietary patterns to be able to compare cross-sectionally the T2D risk between tertiles at the end of the intervention.

### Clinical assessment

Anthropometric measurements, oral glucose tolerance test (OGTT), and laboratory methods used in the T2D-GENE study have been described in detail earlier [41]. We defined T2D as FPG  ≥ 7.0 mmol/l, 2 h PG  ≥ 11.1 mmol/l, or HbA1c  ≥ 48 mmol/mol ( ≥ 6.5%), according to the American Diabetes Association diagnostic criteria [31], or T2D diagnosed at health care. Age was calculated by the participants’ birth date and BMI was measured from height and weight measured at each laboratory visit. Leisure time exercise (at least 2 times per week = “physically active”; none, little or only in context of other hobbies = “physically inactive”), current smoking (yes/no), and total grams of weekly alcohol consumption (calculated by study personnel from questions measuring different types of alcoholic drinks per week) were asked using a self-administered questionnaire during each laboratory visit.

### Genotyping and genetic risk groups

We performed genotyping using either HumanOmniExpress BeadChip-12v1 (Illumina, San Diego, CA, USA; 733 202 markers) or HumanExome-12v1.1 Beadchip (Illumina, 247 870 markers), as described in detail earlier [32]. The non-weighted GRS included the sum of 76 genetic variants that were associated with T2D risk up until 2016 when the recruitment for the T2D-GENE study began [33, 34]. All participants belonged either to the lowest or the highest GRS tertile (GRS ≤ 76 or  ≥ 80). The study participants and staff were blinded to the genetic risk of participants.

### Statistical analyses

We used SPSS (version 29, IBM Corp., Armonk, NY, USA) in the statistical analysis. A paired-samples *t*-test was used to evaluate the effect of lifestyle intervention on participants’ FFQ scores, and independent samples *t*-test to compare the FFQ scores between the intervention and the control groups at the end of the intervention. We used chi-square test for independence to compare categorical baseline characteristics, fat choice differences between the study groups at the end of the intervention and incidence T2D by dietary pattern tertiles. For categorical spread for bread and cooking fat choice changes during the intervention, related samples McNemar change test was conducted.

We assessed the association of dietary patterns and the risk of T2D, and interaction between dietary patterns and T2D risk, using binary logistic regression analysis. In these analyses we pooled the intervention and control groups with end-of-intervention dietary data and re-grouped them based on their end-of-intervention dietary pattern consumption into tertiles (low, medium and high consumption of given dietary pattern) (*n* = 883). We performed the analyses using an unadjusted model (model 1), and by controlling for age, leisure time exercise (in two categories), current smoking, and total alcohol consumption (g/week) (model 2), and lastly by controlling for BMI in addition to previously controlled confounding factors (model 2 + BMI = model 3). In the models, we have used baseline covariate values for variables with unchanged values during the intervention and end-of-intervention values for covariates with statistically significant changes during the three years. We also tested how controlling for the treatment arm (intervention or control group) affected the T2D odds ratios (OR).

To test whether genetic risk modifies the effect of diet in the development of T2D, we further stratified the participants into low and high GRS groups and repeated the binary logistic regression analyses.

There is some variation in the sample sizes between the statistical analyses. This was due to missing data in any individual FFQ variable either at baseline or at year 3, or in single covariates used in adjusted models. The baseline characteristic comparison (Table 1) includes all participants with baseline characteristic data available (*n* = 547 for intervention and *n* = 336 for control group). All the between-group analyses comparing intervention and control groups at year 3 (without comparisons to baseline) were analysed with full sample size available for year 3 dietary variables (*n* = 547 for intervention and *n* = 336 for control group). For T2D risk analyses, we have included participants (*n* = 883) with year 3 dietary pattern data (i.e. data for all foods included in the patterns) available. For the paired analyses comparing baseline and year 3 dietary data, we were only able to include data from the participants with both baseline and year 3 available (*n* = 465 for intervention group, except *n* = 464 for analyses of spread for bread and cooking fat, and *n* = 140 for control group).

We have addressed the rate of type I error from multiple comparisons with Benjamini–Hochberg FDR 5% corrections for results except for T2D incidence comparisons (Table 5). Original *p*-values were ranked by their ascending order and each *p*-value was assigned with Benjamini–Hochberg critical value [(individual *p*-value rank/number of tests) × 0.05]. *P*-values that are smaller than their FDR critical value are considered statistically significant.

## Results

### Changes in food choices

By the end of the 3 year intervention, the intervention group reported lower consumption of several foods high in saturated fat and salt, such as sausage dishes and sausages, sausage cutleries, cheeses with > 17% fat, savoury pies and pastries, unsweetened yoghurts with > 1% fat (all *p* < 0.001) and sweetened yoghurts with > 1% fat (*p* = 0.023), and lower consumption of low-fibre cereal products such as refined pasta and rice, low-fibre breads, buns and bun-based pies (all *p* < 0.001), and low-fibre porridges (*p* = 0.023) as compared to baseline (Table 2). The intervention group also reported higher consumption of whole grain pasta and rice, whole grain porridges, breakfast cereals and muesli (all *p* < 0.001), and low-fat (≤ 17% fat) cheeses (*p* = 0.027), fresh and frozen berries (*p* = 0.012), and poultry (*p* = 0.007).

The control group only reported changes in individual foods during the study period: decreased consumption of low-fat cheeses (*p* < 0.001), fruit and berry juices and sour cream-based salad dressings (both *p* = 0.002).

The differences in food consumption frequencies between the intervention and control groups at year 3 were in line with the dietary targets of the intervention (Table 2). As compared to the controls, the intervention group reported more frequent consumption of fresh salad and fresh vegetables, fruits, fresh or frozen berries, whole grain porridges, fish and fish dishes, oil-based salad dressing or oil with vegetables (all *p* ≤ 0.001), chicken and chicken dishes (*p* = 0.003), boiled side vegetables (*p* = 0.019), and whole grain pasta and rice (*p* = 0.005). They also reported lower consumption of non-recommended foods such as sausages and sausage dishes, sausage cutleries, refined pasta and rice, low-fibre porridges, white breads, cheeses with > 17% fat, sweet cookies/biscuits, and sweet and savoury pastries (all *p* < 0.001), meat and meat dishes (*p* = 0.002), and yoghurts with > 1% fat (unsweetened and sweetened, *p* = 0.013 and *p* < 0.001, respectively).

The intervention group reported lower use of low-fat spreads as their typical spread for bread during the study and there was a significant difference as compared to the control group at year 3 (Table 3). Also, the reported use of butter or a spread with 60–80% fat but not meeting the Heart Symbol criteria (max. 30% saturated fatty acids of total fat and max. 1% salt) decreased in the intervention group (butter users 2.5% at baseline vs. 0.4% at year 3, *p* = 0.008; and users for spreads not meeting the Heart Symbol criteria with 60–80% fat 24.4% vs. 8.8%, *p* < 0.001). The reported use of recommended spreads meeting the Heart Symbol criteria with fat content 55–80% increased in the intervention group during the study (33.3% users at baseline vs. 65.5% at year 3, *p* < 0.001), and were significantly more common in the intervention group as compared to the control group at year 3 (*p* < 0.001). The Heart Symbol is widely used and recognized among the Finnish consumers [35].

**Table 3:** Consumption of typical fat spread on bread and cooking fat used in the intervention and the control groups (proportion of users)

		Intervention group (*n* = 464)			Control group (*n* = 140)			
		Baseline (% of participants)	Year 3 (% of participants)	*p*-value (change over time)^1^	Baseline (% of participants)	Year 3 (% of participants)	*p*-value (change over time)^1^	*p*-value (difference between groups at year 3)^2^
*Typical fat spread used on bread*								
	None	5.1	4.2	0.170	4.8	6.3	0.289	0.175
	Spread meeting the HS criteria* and ≤ 40% fat	18.1	11.3	0.002^†^	22.8	22.3	0.417	< 0.001^†^
	Spread meeting the HS criteria* and 55–80% fat	33.3	65.6	< 0.001^†^	26.2	26.8	0.868	< 0.001^†^
	Spread not meeting the HS criteria* and < 60% fat	5.0	0.7	0.002^†^	9.7	3.9	0.007^†^	< 0.001^†^
	Spread not meeting the HS criteria* and 60–80% fat	24.4	8.8	< 0.001^†^	26.9	26.8	0.832	< 0.001^†^
	Butter	2.5	0.4	0.008^†^	2.1	1.2	0.625	0.147
	Spread with plant sterols and ≤ 40% fat	5.3	2.2	0.009^†^	2.8	5.1	1.000	0.020^†^
	Spread with plant sterols and ≥ 50% fat	6.3	6.8	1.000	4.8	7.7	0.774	0.585
*Typical fat used in cooking*								
	Vegetable oil or liquid vegetable fat product	61.1	67.8	0.011^†^	55.3	54.5	0.874	< 0.001^†^
	Vegetable fat with 60–80% fat	6.6	13.2	< 0.001^†^	7.1	10.7	0.238	0.281
	Baking margarine	0.8	0.5	1.000	0.7	1.5	1.000	0.153
	Butter-vegetable fat mixture	18.7	10.8	0.001^†^	23.4	19.3	0.596	< 0.001^†^
	Butter	10.4	4.8	< 0.001^†^	13.5	11.9	1.000	< 0.001^†^
	Fat with plant sterols	1.7	1.8	1.000	0	1.5	1.000	0.704
	None	0	0.5	0.250	0	0.3	1.000	0.590
	No cooking at home	0.8	0.5	1.000	0	0.3	1.000	0.590

^1^Related samples McNemar change test, ^2^Chi-square test (intervention group *n* = 547, control group  = 336). *The Heart Symbol criteria for fat spreads: max. 30% saturated fatty acids of total fat and max. 1% salt. ^†*n*^Statistically significant after false discovery rate adjustment

The intervention group also reported higher use of vegetable oils or liquid vegetable fat products, or 60–80% vegetable fat as their cooking fat, during the study and there was a significant difference than the control group at year 3 (Table 3). The reported use of butter or butter-vegetable fat mixture were significantly lower in the intervention group as compared to the control group at the end of the study (*p* < 0.001 for both). The use of vegetable oil or liquid vegetable fat product was significantly higher in the intervention group as compared to the control group at year 3 (*p* < 0.001).

The control group reported lower use of spreads not meeting the Heart Symbol criteria with < 60% fat (Table 3) as compared to baseline. No other changes in bread spreads were observed in the control group. There were no changes in the typical cooking fats among the controls.

### Changes in dietary patterns

When we looked at pooled food consumption by dietary patterns, we found that the intervention group both increased their reported consumption of healthy dietary pattern and decreased their reported consumption of unhealthy dietary pattern (both patterns *p* < 0.001) (Table 4). The control group also reported lower consumption of unhealthy pattern as compared to baseline (*p* = 0.002). There was a significant difference at healthy pattern already at baseline favoring the intervention group. Still, the intervention group consumed healthy dietary pattern more frequently and unhealthy dietary pattern more rarely as compared to the control group at the end of the intervention (*p* < 0.001 for both patterns).

**Table 4:** Total scores for food consumption frequency of healthy and unhealthy dietary patterns in the intervention and the control groups at baseline and end of the intervention (year 3)

	Intervention group (*n* = 465)			Control group (*n* = 140)			
	Baseline, mean (SD)	Year 3, mean (SD)	*p*-value (change in time)^3^	Baseline, mean (SD)	Year 3, mean (SD)	*p*-value (change in time)^3^	*p*-value (difference between groups at year 3)^4^
Healthy dietary pattern^1^, *	47.5 (7.93)	48.7 (7.13)	< 0.001^†^	45.0 (7.82)	43.9 (7.77)	0.046	< 0.001^†^
Unhealthy dietary pattern^2^	40.6 (8.52)	35.2 (7.66)	< 0.001^†^	41.5 (8.88)	39.6 (7.72)	0.002^†^	< 0.001^†^

^3^paired samples *t*-test, ^4^independent samples *t*-test (intervention group *n* = 547, control group *n* = 336). *Statistically significant difference between intervention and control group at baseline (with false discovery rate adjustment). ^†^Statistically significant after false discovery rate adjustment

^1^ Fresh salad, fresh vegetables; fresh or frozen berries; boiled side vegetables; fruits; oil-based salad dressing or oil with vegetables; fish and fish dishes; chicken, turkey and chicken dishes; unsweetened or artificially sweetened yoghurt (including dairy-, oat-, soy- and rice-based products), quark, Nordic sour milk, or skyr (≤ 1% fat); vegetable dishes; whole grain porridges; whole grain pasta or rice; low-fat cheeses (fat ≤ 17%); boiled or mashed potatoes

^2^ Fried potatoes or French fries; sausage dishes, sausages; hamburgers; pizza; refined pasta or rice; other sweet pastries; sausage cutleries; other candy; savory pies and pastries; savory snacks; ice cream or puddings; French roll, baguette, or other white bread; sweet cookies, biscuits; meat dishes; ready-meals; cheeses with > 17% fat; sweetened yoghurt (including dairy-, oat-, soy- and rice-based products), quark, or Nordic sour milk (> 1% fat); sour cream-based salad dressing

### Risk for T2D

The T2D incidence varied by dietary pattern tertiles, grouped by the end-of-intervention consumption, in all participants and in those in the high GRS group (Table 5).

**Table 5:** Incident T2D by end-of-intervention dietary pattern tertiles (n incident T2D/n total group size)

	Lowest tertile	Middle tertile	Highest tertile	*p*-value (difference between tertiles)^3^
Healthy dietary pattern^1^				
All participants	27/315 (8.6%)	18/302 (6.0%)	9/266 (3.4%)	0.034
Low GRS	11/178 (6.2%)	5/153 (3.2%)	5/132 (3.8%)	0.397
High GRS	16/137 (11.7%)	13/149 (8.7%)	4/134 (3.0%)	0.026
Unhealthy dietary pattern^2^				
All participants	10/312 (3.2%)	15/280 (5.4%)	29/291 (10.0%)	0.002
Low GRS	7/160 (4.4%)	8/136 (5.9%)	6/167 (3.6%)	0.631
High GRS	3/152 (2.0%)	7/144 (4.9%)	23/124 (18.5%)	< 0.001

GRS = genetic risk score for T2D, ^3^chi-square test for independence

^1^ Fresh salad, fresh vegetables; fresh or frozen berries; boiled side vegetables; fruits; oil-based salad dressing or oil with vegetables; fish and fish dishes; chicken, turkey and chicken dishes; unsweetened or artificially sweetened yoghurt (including dairy-, oat-, soy- and rice-based products), quark, Nordic sour milk, or skyr (≤ 1% fat); vegetable dishes; whole grain porridges; whole grain pasta or rice; low-fat cheeses (fat ≤ 17%); boiled or mashed potatoes

^2^ Fried potatoes or French fries; sausage dishes, sausages; hamburgers; pizza; refined pasta or rice; other sweet pastries; sausage cutleries; other candy; savory pies and pastries; savory snacks; ice cream or puddings; French roll, baguette, or other white bread; sweet cookies, biscuits; meat dishes; ready-meals; cheeses with > 17% fat; sweetened yoghurt (including dairy-, oat-, soy- and rice-based products), quark, or Nordic sour milk (> 1% fat); sour cream-based salad dressing

Both healthy and unhealthy end-of-intervention dietary patterns were associated with the risk of T2D in all participants (Tables 6 and 7). The healthy pattern was associated with decreased risk of T2D (OR 0.62 (95% CI 0.43; 0.89), and the unhealthy pattern was associated with increased risk (OR 1.85 (95% CI 1.29; 2.65)). After controlling the results for confounding factors (age, BMI, leisure time exercise, smoking and alcohol consumption), the results remained significant for both healthy and unhealthy patterns (OR 0.67 (95% CI 0.46; 0.97) and OR 1.82 (95% CI 1.26; 2.62), respectively).

**Table 6:** Type 2 diabetes risk for end-of-intervention healthy dietary pattern tertiles

		Odds ratio (95% CI)	*p*-value
All participants			
	Model 1 (*n* = 883)	0.62 (0.43; 0.89)	0.010
	Model 2 (*n* = 875)	0.66 (0.46; 0.96)	0.029
	Model 3 (*n* = 874)	0.67 (0.46; 0.97)	0.034
Low GRS			
	Model 1 (*n* = 463)	0.74 (0.42; 1.29)	0.285
	Model 2 (*n* = 459)	0.80 (0.44; 1.44)	0.447
	Model 3 (*n* = 458)	0.81 (0.45; 1.47)	0.490
High GRS			
	Model 1 (*n* = 420)	0.53 (0.33; 0.86)	0.010
	Model 2 (*n* = 416)	0.56 (0.34; 0.91)	0.020
	Model 3 (*n* = 416)	0.57 (0.35; 0.93)	0.024

Model 1 = unadjusted values; Model 2 = adjusted with age (years at baseline), exercise at leisure time (in two categories, baseline), smoking (in two categories, baseline), and total alcohol consumption (g/week, year 3); Model 3: Model 2 + BMI (kg/m^2^, year 3). GRS = genetic risk score for T2D

**Table 7:** Type 2 diabetes risk for end-of-intervention unhealthy dietary pattern tertiles

		Odds ratio (95% CI)	*p*-value
All participants			
	Model 1 (*n* = 883)	1.85 (1.29; 2.65)	< 0.001
	Model 2 (*n* = 875)	1.79 (1.25; 2.56)	0.002
	Model 3 (*n* = 874)	1.82 (1.26; 2.62)	0.001
Low GRS			
	Model 1 (*n* = 463)	0.91 (0.54; 1.54)	0.726
	Model 2 (*n* = 459)	0.91 (0.54; 1.54)	0.734
	Model 3 (*n* = 458)	0.92 (0.54; 1.57)	0.756
High GRS			
	Model 1 (*n* = 420)	3.69 (2.08; 6.53)	< 0.001
	Model 2 (*n* = 416)	3.56 (2.00; 6.35)	< 0.001
	Model 3 (*n* = 416)	3.72 (2.07; 6.69)	< 0.001

Model 1 = unadjusted values; Model 2 = adjusted with age (years at baseline), exercise at leisure time (in two categories, baseline), smoking (in two categories, baseline), and total alcohol consumption (g/week, year 3); Model 3: Model 2 + BMI (kg/m^2^, year 3). GRS = genetic risk score for T2D

There was a significant interaction between the unhealthy dietary pattern and GRS (*p* < 0.001) but not for the healthy dietary pattern and GRS (*p* = 0.386). We further stratified the analyses by the genetic risk score. The differences in the risk for T2D were apparent among the participants in the highest GRS group (OR 0.53 (95% CI 0.33; 0.86) for the healthy pattern tertiles, and OR 3.69 (95% CI 1.08; 6.53) for the unhealthy dietary pattern tertiles) (Tables 6 and 7). The results remained significant after controlling for the confounding factors. There were no associations between dietary patterns and the risk of T2D in the low GRS group after the GRS stratification.

Once we controlled the analyses for the treatment arm (intervention or control), the results remained significant for the unhealthy dietary pattern for all participants (OR 1.63, 95% CI 1.12; 2.38, *p* = 0.011) and for those in the high GRS group (OR 3.47, 95% CI 1.92; 6.29, *p* < 0.001). Other associations between the diet and T2D lost their statistical significance (not reported here).

## Discussion

This was the first study to investigate the dietary changes in the T2D-GENE intervention group as compared to the control group, and within the control group. The 3 year lifestyle intervention with group-based and online counselling through a web portal successfully supported the prediabetic participants to healthier food choices. The intervention group reported increased consumption frequency of healthy dietary pattern and individual whole-grain products, berries, chicken and chicken dishes, low-fat cheeses, and vegetable oils. They reduced their reported consumption frequency of several foods high in saturated fat, salt and/or sugar such as sausages and sausage dishes, meat and meat dishes, savory snacks and pastries, cookies, ice cream, and fatty cheeses, and butter in cooking as well as the overall unhealthy dietary pattern. This was shown both in comparison to the baseline diet of the intervention group and by comparing the diet between the intervention and control groups at the end of the study. The changes in food choices found in this study are in line with the dietary targets of the T2D-GENE study, i.e. the Nordic nutrition recommendations [29, 36].

The control group also reported a few changes including decreased consumption of low-fat cheeses, fruit and berry juices and sour cream-based salad dressings. When combined into dietary patterns, the control group also decreased their reported consumption for unhealthy foods. The intervention group reported higher scores for healthy dietary pattern and lower scores for unhealthy dietary pattern at year 3 as compared to the control group.

Our results are supported by findings in several previous studies concluding that diet can be altered with professionally led lifestyle counselling [2, 3, 6–10, 13–15]. Several previous studies have applied individual lifestyle counselling [2, 3, 6–10]. Our study demonstrates that the improvement in dietary habits can be obtained also by a group-based setting.

Our study also shows an association between dietary patterns and the risk of T2D: the healthy diet was associated with decreased risk of T2D as the unhealthy diet associated with increased the risk of T2D when all participants were included in statistical analysis. The results remained statistically significant after the adjustment for confounding factors.

When we stratified the participants by their GRS, we found that the association between the diet and T2D was statistically significant in the participants in the highest genetic risk tertile both in the participants on healthy and unhealthy diet, with lower T2D risk with higher healthy dietary pattern consumption and higher T2D risk with higher unhealthy pattern consumption. There was a statistically significant interaction between the unhealthy diet and the genetic risk in the T2D risk (*p* < 0.001). This suggests that an unhealthy diet is especially harmful for those carrying several genetic risk variants for T2D.

The results showing that diet affects especially those with the highest genetic risk for T2D is a rather novel finding. We have previously shown in the T2D-GENE study that the overall lifestyle intervention was especially beneficial in the highest genetic risk group [26]. This previous publication from the T2D-GENE study compared the incident T2D between the intervention and control groups and whether the participation in the lifestyle program overall affected the risk of T2D. Previous study also regarded T2D risk in relation to the GRS. The changes in diet were assessed in Lankinen et al*.* 2024 but based on food records within the intervention group only. We have also previously reported cross-sectional results from the METSIM study showing that the healthy dietary pattern, but not the unhealthy pattern, was associated with lower risk for hyperglycemia in the highest consumption tertile as compared to the lowest tertile in both low and high GRS groups [25]. The population, however, included also normoglycemic Finnish men and we investigated the risk for hyperglycemia (prediabetes or T2D) instead of risk for T2D only which may explain some differences in the results.

A few previous intervention studies have investigated a single gene variant, transcription factor 7-like 2, and shown that the added T2D risk by the gene polymorphism could be invalidated by successful lifestyle changes [37, 38]. Additionally, there are some epidemiological studies showing an interaction between the unhealthy, Western-type diet and GRS for T2D [20, 21]. However, the evidence on the interaction between diet and genetic risk in the development of T2D remains conflicting [39].

The strength of this study comes from its interventional setting and the inclusion of both intervention and control arms. The inclusion of genetic risk is novel. We were able to assess the interaction between the diet and the genetic risk score in the risk of T2D. The T2D-GENE study evaluates the efficacy of a group-based lifestyle program in all-IFG population, most having isolated IFG, and the utilisation of group-based and online counselling, which adds novelty to the existing literature on lifestyle prevention of T2D.

Our study has some limitations. We included in our study only middle-aged and elderly men having Caucasian background. Therefore, the generalisability of the results to women or other ethnic and age groups can be limited. The allocation to the intervention and control groups was non-randomised. The control group participating in the 3-year laboratory visit was smaller than anticipated and there is an imbalance between the study group sizes. Especially with paired comparisons, the number of FFQs available for the control group at both baseline and year 3 is low (*n* = 140). The controls were significantly older, and their consumption of healthy dietary pattern was lower at baseline as compared to the intervention group. It may be that those willing to participate in a lifestyle program were already more interested in improving their dietary habits. This may cause bias to our results. The self-reported FFQ can be prone to misreporting [38] even though the filling was professionally guided. FFQs have been used and shown valid in elderly populations earlier [40–42]. The reported consumption of unhealthy diet was reduced also in the control group, not only in the intervention group. Still, there is a significant difference between the study groups in the consumption of unhealthy dietary pattern at the end-of-intervention.

## Conclusions

Our study shows that a 3 year group-based lifestyle intervention, utilising online counselling, helps to improve the diet among middle-aged to elderly men receiving lifestyle counselling. Healthy and unhealthy dietary patterns were associated with the risk of T2D, especially in the individuals having a high genetic risk. This highlights the importance of the prevention of T2D with dietary changes in the individuals with prediabetes and a high genetic risk for T2D. Our results can be used in health care and decision making to improve prevention of T2D. To identify persons with high genetic risk for T2D, either genetic testing or inquiries of family history for T2D should be used.

## Supplementary Information

Below is the link to the electronic supplementary material.

Supplementary Material 1

## Data Availability

Restrictions apply to the availability of data generated or analysed during this study to preserve the confidentiality of the participants. The corresponding author will, on request, detail the restrictions and any conditions under which access to some data may be provided.

## Abbreviations

BMI: Body mass index
CI: Confidence interval
FDR: False discovery rate
FFQ: Food frequency questionnaire
FPG: Fasting plasma glucose
GRS: Genetic risk score
IFG: Impaired fasting glucose
IGT: Impaired glucose tolerance
Matsuda ISI: Matsuda insulin sensitivity index
METSIM: Metabolic syndrome in men
OGTT: Oral glucose tolerance test
OR: Odds ratio
PCA: Principal component analysis
PG: Plasma glucose
SD: Standard deviation
T2D: Type 2 diabetes

## References

[1] International Diabetes Federation IDF Diabetes Atlas - 10th edition 35914061

[2] Tuomilehto J, Lindström J, Eriksson JG et al (2001) Prevention of type 2 diabetes mellitus by changes in lifestyle among subjects with impaired glucose tolerance. N Engl J Med 344:1343–1350. 10.1056/NEJM200105033441801 11333990 10.1056/NEJM200105033441801

[3] Knowler WC, Barrett-Connor E, Fowler SE et al (2002) Reduction in the incidence of type 2 diabetes with lifestyle intervention or metformin. N Engl J Med 346:393–403. 10.1056/NEJMoa012512 11832527 10.1056/NEJMoa012512 PMC1370926

[4] Fuchsberger C, Flannick J, Teslovich TM et al (2016) The genetic architecture of type 2 diabetes. Nature 536:41–47. 10.1038/nature18642 27398621 10.1038/nature18642 PMC5034897

[5] Laakso M (2019) Biomarkers for type 2 diabetes. Mol Metab 27:S139–S146. 10.1016/j.molmet.2019.06.016 10.1016/j.molmet.2019.06.016 PMC6768493 31500825

[6] Pan X-R, Li G-W, Hu Y-H et al (1997) Effects of diet and exercise in preventing NIDDM in people with impaired glucose tolerance: the Da Qing IGT and Diabetes Study. Diabetes Care 20:537–544. 10.2337/diacare.20.4.537 9096977 10.2337/diacare.20.4.537

[7] Penn L, White M, Oldroyd J et al (2009) Prevention of type 2 diabetes in adults with impaired glucose tolerance: the European Diabetes Prevention RCT in Newcastle upon Tyne. UK BMC Public Health 9:342. 10.1186/1471-2458-9-342 19758428 10.1186/1471-2458-9-342 PMC2760530

[8] Ramachandran A, Snehalatha C, Mary S et al (2006) The Indian Diabetes Prevention Programme shows that lifestyle modification and metformin prevent type 2 diabetes in Asian Indian subjects with impaired glucose tolerance (IDPP-1). Diabetologia 49:289–297. 10.1007/s00125-005-0097-z 16391903 10.1007/s00125-005-0097-z

[9] Saito T, Watanabe M, Nishida J et al (2011) Lifestyle modification and prevention of type 2 diabetes in overweight Japanese with impaired fasting glucose levels: a randomized controlled trial. Arch Intern Med 171:1352–1360. 10.1001/archinternmed.2011.275 21824948 10.1001/archinternmed.2011.275

[10] Kosaka K, Noda M, Kuzuya T (2005) Prevention of type 2 diabetes by lifestyle intervention: a Japanese trial in IGT males. Diabetes Res Clin Pract 67:152–162. 10.1016/j.diabres.2004.06.010 15649575 10.1016/j.diabres.2004.06.010

[11] Thankappan KR, Sathish T, Tapp RJ et al (2018) A peer-support lifestyle intervention for preventing type 2 diabetes in India: a cluster-randomized controlled trial of the Kerala Diabetes Prevention Program. PLoS Med 15:e1002575. 10.1371/journal.pmed.1002575 29874236 10.1371/journal.pmed.1002575 PMC5991386

[12] Davies MJ, Gray LJ, Troughton J et al (2016) A community based primary prevention programme for type 2 diabetes integrating identification and lifestyle intervention for prevention: the let’s prevent diabetes cluster randomised controlled trial. Prev Med 84:48–56. 10.1016/j.ypmed.2015.12.012 26740346 10.1016/j.ypmed.2015.12.012

[13] Weber MB, Ranjani H, Staimez LR et al (2016) The stepwise approach to diabetes prevention: results from the D-CLIP randomized controlled trial. Diabetes Care 39:1760–1767. 10.2337/dc16-1241 27504014 10.2337/dc16-1241 PMC5033082

[14] Duijzer G, Haveman-Nies A, Jansen SC et al (2017) Effect and maintenance of the SLIMMER diabetes prevention lifestyle intervention in Dutch primary healthcare: a randomised controlled trial. Nutr Diabetes 7:e268. 10.1038/nutd.2017.21 28481335 10.1038/nutd.2017.21 PMC5518803

[15] Bo S, Ciccone G, Baldi C et al (2007) Effectiveness of a lifestyle intervention on metabolic syndrome. A randomized controlled trial. J Gen Intern Med 22:1695–1703. 10.1007/s11606-007-0399-6 17922167 10.1007/s11606-007-0399-6 PMC2219825

[16] Merino J, Guasch-Ferré M, Ellervik C et al (2019) Quality of dietary fat and genetic risk of type 2 diabetes: individual participant data meta-analysis. BMJ 366:l4292. 10.1136/bmj.l4292 31345923 10.1136/bmj.l4292 PMC6652797

[17] Ericson U, Hindy G, Drake I et al (2018) Dietary and genetic risk scores and incidence of type 2 diabetes. Genes Nutr 13:13. 10.1186/s12263-018-0599-1 29796113 10.1186/s12263-018-0599-1 PMC5956794

[18] Zhang S, Stubbendorff A, Olsson K et al (2023) Adherence to the EAT-lancet diet, genetic susceptibility, and risk of type 2 diabetes in Swedish adults. Metabolism 141:155401. 10.1016/j.metabol.2023.155401 36682448 10.1016/j.metabol.2023.155401

[19] Langenberg C, Sharp SJ, Franks PW et al (2014) Gene-lifestyle interaction and type 2 diabetes: the EPIC interact case-cohort study. PLoS Med 11:e1001647. 10.1371/journal.pmed.1001647 24845081 10.1371/journal.pmed.1001647 PMC4028183

[20] Qi L, Cornelis MC, Zhang C et al (2009) Genetic predisposition, Western dietary pattern, and the risk of type 2 diabetes in men123. Am J Clin Nutr 89:1453–1458. 10.3945/ajcn.2008.27249 19279076 10.3945/ajcn.2008.27249 PMC2676999

[21] Kim DS, Kim BC, Daily JW, Park S (2018) High genetic risk scores for impaired insulin secretory capacity doubles the risk for type 2 diabetes in Asians and is exacerbated by western-type diets. Diabetes Metab Res Rev 34:e2944. 10.1002/dmrr.2944 10.1002/dmrr.2944 29048714

[22] Li SX, Imamura F, Schulze MB et al (2018) Interplay between genetic predisposition, macronutrient intake and type 2 diabetes incidence: analysis within EPIC-InterAct across eight European countries. Diabetologia 61:1325–1332. 10.1007/s00125-018-4586-2 29549418 10.1007/s00125-018-4586-2 PMC6445347

[23] Sonestedt E, Lyssenko V, Ericson U et al (2012) Genetic variation in the glucose-dependent insulinotropic polypeptide receptor modifies the association between carbohydrate and fat intake and risk of type 2 diabetes in the Malmo Diet and Cancer cohort. J Clin Endocrinol Metab 97:E810–818. 10.1210/jc.2011-2444 22399504 10.1210/jc.2011-2444

[24] Jia X, Xuan L, Dai H et al (2021) Fruit intake, genetic risk and type 2 diabetes: a population-based gene–diet interaction analysis. Eur J Nutr 60:2769–2779. 10.1007/s00394-020-02449-0 33399975 10.1007/s00394-020-02449-0 PMC8275558

[25] Tolonen U, Lankinen M, Laakso M, Schwab U (2024) Healthy dietary pattern is associated with lower glycemia independently of the genetic risk of type 2 diabetes: a cross-sectional study in Finnish men. Eur J Nutr 63:2521–2531. 10.1007/s00394-024-03444-5 38864868 10.1007/s00394-024-03444-5 PMC11490453

[26] Lankinen MA, Nuotio P, Kauppinen S et al (2024) Effects of genetic risk on incident type 2 diabetes and glycemia: the T2D-GENE lifestyle intervention trial. J Clin Endocrinol Metab. 10.1210/clinem/dgae422 38888187 10.1210/clinem/dgae422 PMC11651687

[27] Laakso M, Kuusisto J, Stančáková A et al (2017) The metabolic syndrome in men study: a resource for studies of metabolic and cardiovascular diseases. J Lipid Res 58:481–493. 10.1194/jlr.O072629 28119442 10.1194/jlr.O072629 PMC5335588

[28] Schwab U, Lankinen M, Laakso M (2021) Effect of lifestyle intervention on the risk of incident diabetes in individuals with impaired fasting glucose and low or high genetic risk for the development of type 2 diabetes in men: a T2D-GENE trial. Food Nutr Res 65:10.29219/fnr.v65.7721. 10.29219/fnr.v65.7721 10.29219/fnr.v65.7721 PMC8494262 34650392

[29] Schwab U, Lankinen M, Uusitupa M, Laakso M (2023) Lifestyle intervention guided by group and internet-based counseling in the T2D-GENE trial supports its applicability and feasibility. Nutrients 15:1787. 10.3390/nu15071787 37049626 10.3390/nu15071787 PMC10097002

[30] Finnish Institute for Health and Welfare, The National FINRISK study. Questionnaires. https://thl.fi/documents/189940/4850942/finriski2007questionnaire.pdf/ee4e02c0-1cde-4474-9d4d-9e55697007b5

[31] American Diabetes Association (2013) Diagnosis and classification of diabetes mellitus. Diabetes Care 37:S81–S90. 10.2337/dc14-S081 10.2337/dc14-S081 24357215

[32] Stančáková A, Kuulasmaa T, Kuusisto J et al (2017) Genetic risk scores in the prediction of plasma glucose, impaired insulin secretion, insulin resistance and incident type 2 diabetes in the METSIM study. Diabetologia 60:1722–1730. 10.1007/s00125-017-4313-4 28573393 10.1007/s00125-017-4313-4

[33] Mohlke KL, Boehnke M (2015) Recent advances in understanding the genetic architecture of type 2 diabetes. Hum Mol Genet 24:R85–R92. 10.1093/hmg/ddv264 26160912 10.1093/hmg/ddv264 PMC4572004

[34] Zeggini E, Scott LJ, Saxena R et al (2008) Meta-analysis of genome-wide association data and large-scale replication identifies additional susceptibility loci for type 2 diabetes. Nat Genet 40:638–645. 10.1038/ng.120 18372903 10.1038/ng.120 PMC2672416

[35] Lahti-Koski M, Helakorpi S, Olli M et al (2012) Awareness and use of the heart symbol by Finnish consumers. Public Health Nutr 15:476–482. 10.1017/S136898001100187X 21835085 10.1017/S136898001100187X

[36] Nordic Nutrition Recommendations 2023 / Integrating Environmental Aspects. https://pub.norden.org/nord2023-003/. Accessed 17 Aug 2023

[37] Wang J, Kuusisto J, Vänttinen M et al (2007) Variants of transcription factor 7-like 2 (TCF7L2) gene predict conversion to type 2 diabetes in the finnish diabetes prevention study and are associated with impaired glucose regulation and impaired insulin secretion. Diabetologia 50:1192–1200. 10.1007/s00125-007-0656-6 17437080 10.1007/s00125-007-0656-6

[38] Florez JC, Jablonski KA, Bayley N et al (2006) TCF7L2 polymorphisms and progression to diabetes in the diabetes prevention program. N Engl J Med 355:241–250. 10.1056/NEJMoa062418 16855264 10.1056/NEJMoa062418 PMC1762036

[39] Dietrich S, Jacobs S, Zheng J-S et al (2019) Gene-lifestyle interaction on risk of type 2 diabetes: a systematic review. Obes Rev 20:1557–1571. 10.1111/obr.12921 31478326 10.1111/obr.12921 PMC8650574

[40] Jian L, Binns CW, Lee AH (2006) Validity of a food-frequency questionnaire for elderly men in southeast China. Public Health Nutr 9:928–933. 10.1017/PHN2005919 17010259 10.1017/phn2005919

[41] Smith W, Mitchell P, Reay EM et al (1998) Validity and reproducibility of a self-administered food frequency questionnaire in older people. Aust N Z J Public Health 22:456–463. 10.1111/j.1467-842X.1998.tb01414.x 9659773 10.1111/j.1467-842x.1998.tb01414.x

[42] Talegawkar SA, Tanaka T, Maras JE et al (2015) Validation of nutrient intake estimates derived using a semi-quantitative FFQ against 3 day diet records in the baltimore longitudinal study of aging. J Nutr Health Aging 19:994–1002. 10.1007/s12603-015-0659-9 26624210 10.1007/s12603-015-0659-9 PMC6139669

